# Thoracic Endovascular Aortic Repair After Salvage Coronary Artery Bypass Graft for Type A Dissection

**DOI:** 10.1016/j.atssr.2022.09.004

**Published:** 2022-09-20

**Authors:** Yasutaka Yokoyama, Shizuyuki Dohi, Takeshi Kinoshita, Daisuke Endo, Taira Yamamoto, Atsushi Amano, Minoru Tabata

**Affiliations:** 1 Department of Cardiovascular Surgery, Toda Chuo General Hospital, Saitama, Japan; 2 Department of Cardiovascular Surgery, Juntendo University, Tokyo, Japan

## Abstract

Acute aortic dissection (AAD) is a life-threatening condition. It has a high death rate, especially in malperfusion. An 84-year-old man diagnosed with AAD with complete right coronary artery occlusion underwent bypass of only 1 branch of the coronary artery; the chest was closed without treating the AAD. After 2 months, zone 0 thoracic endovascular aortic repair was performed for the AAD. The patient was uneventfully discharged. Treating AAD with complete right coronary artery occlusion for malperfusion first, followed by 2 phases of thoracic endovascular aortic repair for AAD, may be a useful treatment option.

Thoracic endovascular aortic repair (TEVAR) is experimental for the ascending aorta. However, TEVAR could be a last resort if the anatomic conditions are met: appropriate length of the ascending aorta and healthy landing zones.^1^ We report a case of a patient who underwent delayed TEVAR for a rapidly enlarging ascending aorta 2 months after salvage coronary artery bypass grafting (CABG), which was performed for acute pump failure secondary to coronary malperfusion complicated with thrombosed-type acute aortic dissection (AAD).

An 84-year-old man was admitted to our hospital with sudden-onset chest pain. Initial electrocardiography suggested an inferior wall acute myocardial infarction. The patient was immediately referred to the catheterization laboratory for coronary angiography, which revealed eccentric stenosis at the proximal portion of the right coronary artery (RCA) and an intact left coronary artery. The luminal narrowing in the RCA was caused by an extrinsic compression of the vessel wall. Aortography revealed an aortic dissection flap with aortic dilation, indicative of an underlying type A AAD. Urgent computed tomography (CT) confirmed the diagnosis of a type A AAD with RCA ostium involvement (Figure 1A). The patient's hemodynamics were stable, and the false lumen was filled with a small thrombus. There was no significant pericardial fluid retention. The patient was transferred urgently to the operating room for central repair and coronary revascularization. However, the patient’s hemodynamics collapsed rapidly during median sternotomy, and coronary revascularization was completed using the saphenous vein. A distal coronary anastomosis to the posterior descending artery was created under on-pump beating without aortic cross-clamping. The right axillary artery was the inflow site for the graft. Through coronary revascularization, the hemodynamics recovered. Transit-time flow measurement revealed an excellent graft flow (46 mL/min). Thus, only CABG procedure was performed with careful monitoring of the patient for AAD. He was successfully withdrawn from cardiopulmonary bypass and was transferred to the intensive care unit without intra-aortic balloon pumping. He was uneventfully discharged on postoperative day 23 after receiving antihypertensive treatment and rehabilitation without an increase in the ascending aorta diameter on CT. Two months later, follow-up CT revealed a rapid enlargement of the ascending aorta (40-50 mm) and descending aorta (40-55 mm) and recanalization of the false lumen of the ascending and descending aorta (Figure 1B). Considering the general condition of elderly patients, suitable surgical anatomy for stent grafting, and high risk of open redo operation, we chose TEVAR of the ascending and descending aorta as an alternative treatment strategy. Although the ascending aorta had expanded to a maximum diameter of 51 mm, it also had healthy proximal and distal landing zones with adequate diameters (32 mm and 43 mm, respectively) and lengths (22.3 mm and 27.6 mm, respectively). The distance from the left coronary artery to the brachiocephalic trunk was 124.6 mm. The descending aorta also had healthy proximal and distal landing zones with adequate diameters (33 mm and 24 mm) and lengths (41 mm and 29 mm; Figure 2A). The descending aorta was first repaired using a Valiant Captivia thoracic stent graft (Medtronic Vascular). Based on measurements from intravascular ultrasound (Philips Volcano; Figure 2B), we selected a conformable TAG (c-TAG; WL Gore & Associates) for the ascending aorta. The first c-TAG (TGU454510J) was deployed proximal to the orifice of the brachiocephalic artery to cover the intima tear at the ascending aorta under rapid pacing. Next, based on the entrance of the left main trunk, the second c-TAG (TGU454510J) was placed with sufficient overlap with the first one. Final aortography revealed no flow into the false lumen. The patient was uneventfully discharged on postoperative day 5. CT performed 3 years after TEVAR revealed a signiﬁcant enlargement of the true lumen and shrinkage of the false lumen (Figure 1C).

**Figure 1:**
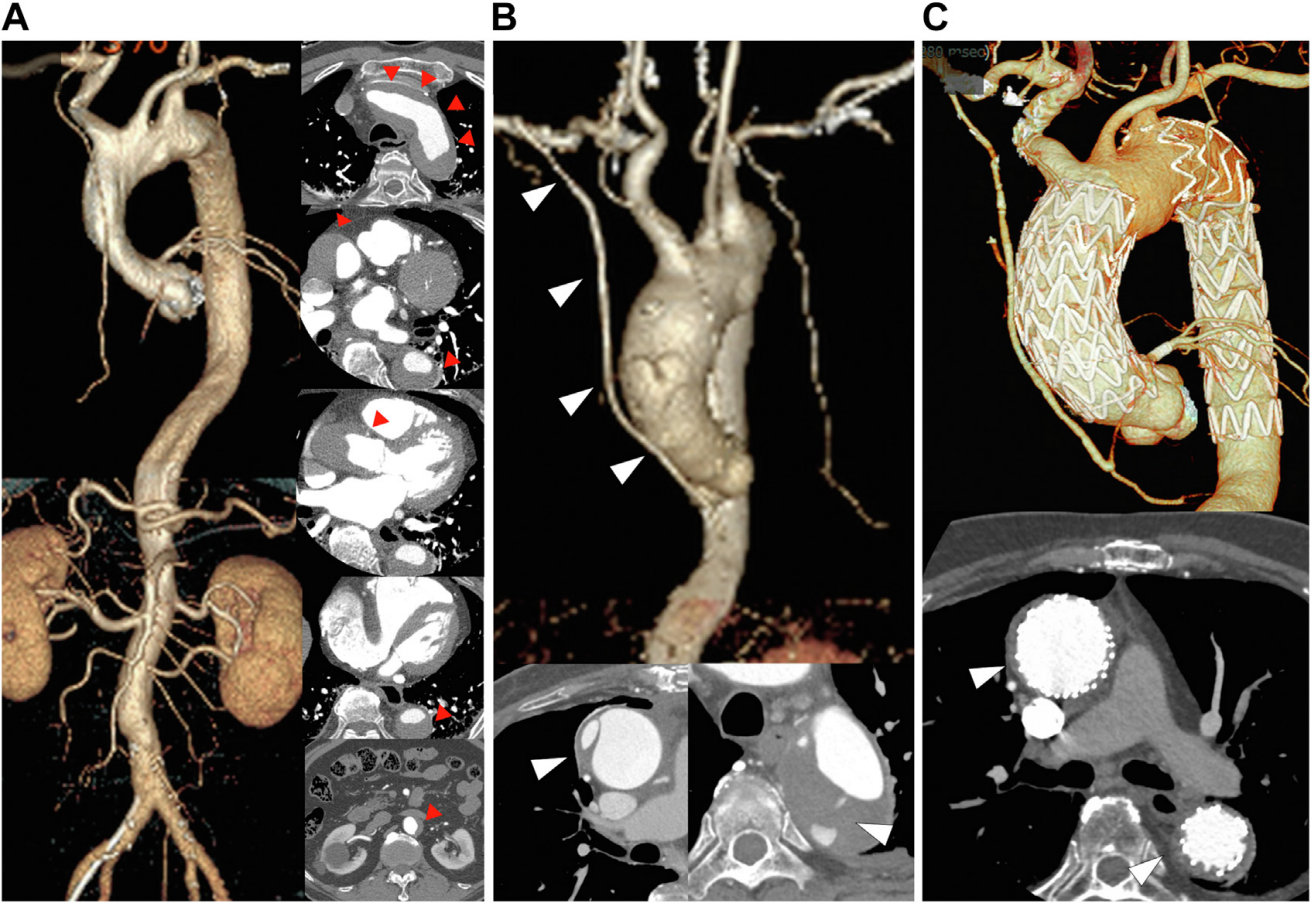
(A) Preoperative contrast-enhanced computed tomography (CT) scan (before coronary artery bypass grafting) showing false lumen thrombosis extending from the ascending aorta to the abdominal aorta, with right coronary artery occlusion (arrowheads). (B) Postoperative contrast-enhanced CT scan (2 months after coronary artery bypass grafting) showing patency of the saphenous vein graft, open false lumen of the ascending aorta, and thrombosed false lumen of the descending aorta. Tears in the ascending and descending aorta (arrowheads). (C) Postoperative contrast-enhanced CT scan (3 years after thoracic endovascular aortic repair) showing complete shrinkage of the false lumens in the ascending and descending aorta and enlargement of the true lumen (arrowheads).

**Figure 2:**
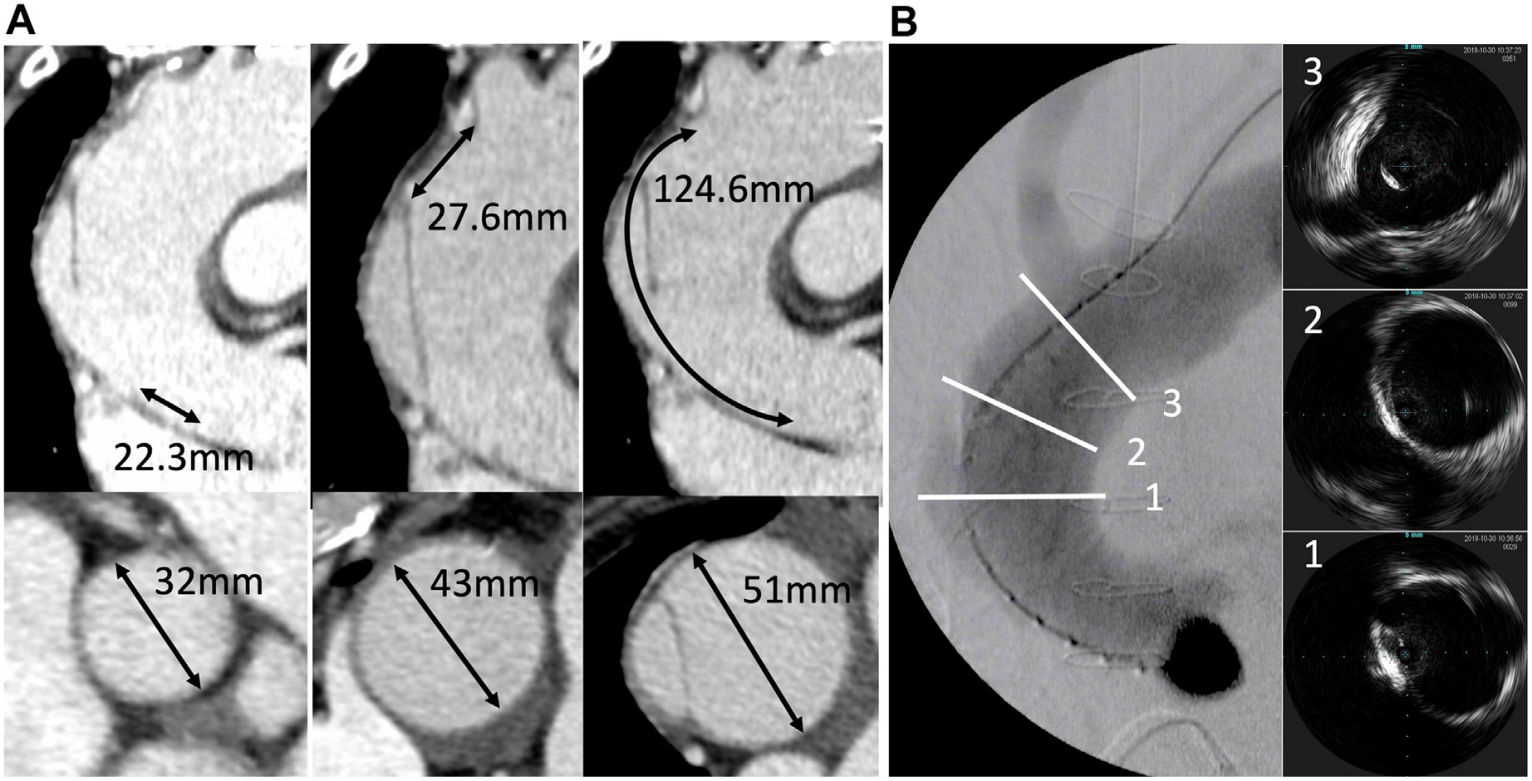
(A) Contrast-enhanced computed tomography scan before thoracic endovascular aortic repair. The proximal diameter and landing zone were 32 mm and 22.3 mm; the distal diameter and landing zone were 43 mm and 27.6 mm; the maximum diameter and length of the greater curvature of the ascending aorta were 51 mm and 124.6 mm, respectively. (B) Intraoperative intravascular ultrasound image (during thoracic endovascular aortic repair) showing (1) an open false lumen in the ascending aorta, (2) distal rounding zone, and (3) brachiocephalic trunk. Length of the ascending aorta >100 mm.

## Comment

Type A AAD with coronary malperfusion is associated with death rates of 21.4% to 39.4%.^2^ Coronary artery dissection is usually managed by replacing the ascending aorta and reconstructing the coronary artery. However, extensive myocardial infarction frequently occurs before surgical restoration of coronary blood flow. Therefore, performing percutaneous coronary intervention before AAD treatment may prevent postoperative low-output syndrome.^3^ However, in our case, percutaneous coronary intervention was unsuccessful. Therefore, myocardial ischemia was resolved by performing CABG procedure because of the prolonged ischemia time and absence of false lumen blood flow in the ascending aorta on preoperative contrast-enhanced CT. There were pros and cons to performing only CABG and observing the patient without performing an aortic repair for the type A AAD. The JCS/JSCVS/JATS/JSVS 2020 guidelines suggest watchful waiting as an alternative treatment option when the false lumen is completely occluded with a thrombus, occupies a small area, and is hemodynamically stable.^4^ Because the hemodynamics of our elderly patient had already collapsed after a complicated acute myocardial infarction, the post-CABG aortic repair risk was deemed extremely high. Furthermore, because his hemodynamics were stable after CABG and his aortic dissection was the thrombus occlusion type, we continued follow-up.

The thrombosed-type aortic dissection was treated with TEVAR within 2 months as contrast-enhanced CT in the outpatient setting revealed rapid enlargement of the false lumen. Two types of TEVAR devices are Zenith (Cook Medical) and c-TAG. Zenith has a long top tip (5 cm) suitable for the apical approach, whereas c-TAG has a short top tip (3 cm) and is ideal for the femoral artery approach. We selected the femoral artery approach to avoid chest reopening; we chose c-TAG to avoid left ventricular injury as reported previously.^5^ Moreover, the 45-mm c-TAG is more suitable than Zenith because it can be used in vessels with a diameter of 34 to 42 mm.

Based on this case, it is unclear whether our treatment strategy of saving the patient with only 1 CABG procedure and TEVAR of the ascending aorta alone in an untreated subacute aortic dissection is a valuable alternative. Further research is required to investigate its usefulness in similar cases.

In conclusion, AAD-associated myocardial ischemia was relieved by CABG procedure alone and closure of the chest without treatment of aortic dissection. Two-stage TEVAR may be a viable rescue option for the remaining aortic dissections.

## References

[1] Plichta R.P., Hughes G.C. (2018). Thoracic endovascular aortic repair for the ascending aorta: experience and pitfalls. J Vis Surg.

[2] Uchida K., Karube N., Minami T. (2018). Treatment of coronary malperfusion in type A acute aortic dissection. Gen Thorac Cardiovasc Surg.

[3] Imoto K., Uchida K., Karube N. (2013). Risk analysis and improvement of strategies in patients who have acute type A aortic dissection with coronary artery dissection. Eur J Cardiothorac Surg.

[4] Ogino H., Iida O., Akutsu K. JCS/JSCVS/JATS/JSVS 2020 guideline on diagnosis and treatment of aortic aneurysm and aortic dissection [in Japanese]. Accessed October 7, 2022. https://www.j-circ.or.jp/cms/wp-content/uploads/2020/07/JCS2020_Ogino.pdf.

[5] Li Z., Lu Q., Feng R. (2016). Outcomes of endovascular repair of ascending aortic dissection in patients unsuitable for direct surgical repair. J Am Coll Cardiol.

